# Prognostic Significance of MSI and EBV Positivity in PD‐L1 Positive Gastric Cancer: A Systematic Review and Meta‐Analysis

**DOI:** 10.1002/cam4.71711

**Published:** 2026-03-19

**Authors:** Fausto Petrelli, Maria Antista, Antonio Ghidini, Valentina Rampulla, Lorenzo Dottorini, Andrea Celotti, Fulvia Milena Cribiu, Barbara Galassi, Ornella Garrone, Alberto Zaniboni, Gianluca Tomasello, Michele Ghidini

**Affiliations:** ^1^ Oncology Unit ASST Bergamo Ovest Treviglio Italy; ^2^ Oncology Unit ASST Crema Crema Italy; ^3^ Oncology Unit Casa di Cura Igea Milano Italy; ^4^ Surgery Unit ASST Bergamo Ovest Treviglio Italy; ^5^ Surgery Unit ASST Cremona Cremona Italy; ^6^ Pathology Unit ASST Bergamo Ovest Treviglio Italy; ^7^ Oncology Unit Fondazione IRCCS Ca' Granda Ospedale Maggiore Policlinico Milano Italy; ^8^ Oncology Unit Fondazione Poliambulanza Brescia Italy

**Keywords:** EBV, gastric cancer, meta‐analysis, MSI, PD‐L1, prognosis

## Abstract

**Background and Aims:**

Microsatellite instability (MSI), programmed death‐ligand 1 (PD‐L1) expression, and Epstein–Barr virus (EBV) positivity are emerging biomarkers in gastric cancer prognosis and treatment selection, particularly in immunotherapy. This review evaluates their prognostic significance through a systematic review and meta‐analysis.

**Methods:**

Relevant studies from PubMed, EMBASE, and the Cochrane Library (January 2010 to December 2024) were analyzed. Studies included assessing MSI, PD‐L1, and EBV status in gastric cancer using immunohistochemistry, PCR, or in situ hybridization, and reported outcomes such as overall survival (OS), disease‐free survival (DFS), or progression‐free survival. Data extraction adhered to PRISMA guidelines, and pooled analyses were conducted using a random‐effects model (DerSimonian‐Laird method). Heterogeneity was assessed using *I* ^2^ statistics and Cochran's *Q* test.

**Results:**

A total of 25 studies involving 6494 patients were reviewed. In localized gastric cancer, MSI‐high status was associated with significantly improved DFS (hazard ratio [HR], 0.42; 95% confidence interval [CI], 0.23–0.75; *p* = 0.004) but showed no significant impact on OS (HR, 0.78; 95% CI, 0.48–1.28; *p* = 0.33) compared to microsatellite stable/PD‐L1‐negative tumors. EBV‐positive/PD‐L1‐positive cancers demonstrated a prognosis similar to EBV‐negative/PD‐L1‐negative cases (OS: HR, 1.08; 95% CI, 0.81–1.45; *p* = 0.59).

**Conclusions:**

In metastatic disease, MSI and EBV status were not associated with significant prognostic effects. MSI and EBV status have minimal prognostic value in gastric cancer, particularly for OS, but are essential for selecting candidates for immune checkpoint inhibitors. Standardizing biomarker evaluation is critical to enhancing their clinical relevance.

## 1 Introduction

Gastric cancer remains a major global health burden, ranking as the fifth most common malignancy and the fourth leading cause of cancer‐related mortality worldwide, with an estimated 1.09 million new cases and 769,000 deaths annually [1]. Despite advances in multimodal treatment approaches, the prognosis for gastric cancer patients remains poor, with 5‐year survival rates ranging from 20% to 40% in most regions [2]. The heterogeneous biology of gastric cancer contributes to variable treatment outcomes, emphasizing the need for precision medicine approaches and reliable prognostic biomarkers [3].

Microsatellite instability (MSI) and programmed death‐ligand 1 (PD‐L1) expression have emerged as significant therapeutic biomarkers in gastric cancer. MSI, resulting from defects in DNA mismatch repair (MMR) genes (MLH1, MSH2, MSH6, PMS2), is found in approximately 8.5%–37% of gastric cancers depending on the population studied and detection methodology [4, 5, 6]. The Cancer Genome Atlas (TCGA) comprehensive molecular characterization of gastric adenocarcinoma identified MSI‐high (MSI‐H) tumors as one of four distinct molecular subtypes, accounting for approximately 22% of cases [7]. MSI‐H tumors possess a high tumor mutational burden and a more immunogenic microenvironment, characterized by increased immune cell infiltration and neoantigen presentation [8].

Epstein–Barr virus (EBV)‐associated gastric cancer represents another molecularly distinct subtype, comprising approximately 9% of gastric adenocarcinomas globally [7]. A comprehensive meta‐analysis by Murphy et al. reported an overall EBV prevalence of 8.7% (95% CI: 7.5%–9.8%) across 70 studies, with notable geographic variation—higher prevalence in the Americas (9.9%) and Europe (9.2%) compared to Asia (8.3%) [9]. EBV‐positive gastric cancers are characterized by extreme CpG island methylator phenotype (CIMP), PIK3CA mutations, and amplification of JAK2, CD274 (PD‐L1), and PDCD1LG2 (PD‐L2) [7].

PD‐L1, expressed on tumor and immune cells, interacts with the PD‐1 receptor on T cells, inhibiting T cell activity and allowing cancer cells to escape immune detection [10]. PD‐L1 expression varies in gastric cancer, with reported prevalence ranging from 25% to 65% depending on the antibody used, scoring system applied, and disease stage [11, 12, 13]. Notably, PD‐L1 expression is often elevated in MSI‐H and EBV‐positive tumors, with positivity rates of approximately 50%–60% in these molecular subtypes compared to 25%–30% in microsatellite stable (MSS) tumors [14, 15, 16].

PD‐L1 has been recognized as a predictive biomarker for immune checkpoint inhibitors (ICIs), showing enhanced response rates in tumors with high PD‐L1 expression and tumor‐infiltrating lymphocytes [17]. Consequently, MSI‐H and PD‐L1‐positive gastric tumors represent potential candidates for immunotherapy, potentially improving outcomes over conventional treatments [18]. A notable correlation exists between MSI and PD‐L1 expression, highlighting the promise of using combination biomarker strategies for anticipating treatment responses.

Research indicates that MSI‐H and EBV‐positive gastric cancers typically exhibit high PD‐L1 expression alongside immune cell infiltration [19, 20]. This suggests that these tumors may evade immune surveillance through the PD‐1/PD‐L1 pathway, positioning them as suitable candidates for immune checkpoint inhibitors. However, while the predictive value of these biomarkers for immunotherapy response is well established, their independent prognostic significance—particularly in patients not receiving immunotherapy—remains controversial.

This meta‐analysis aims to aggregate data on the prognostic significance of MSI, EBV, and PD‐L1 co‐expression to clarify their relationship with clinical outcomes, especially overall survival, in gastric cancer patients treated with surgery and/or conventional chemotherapy.

## 2 Materials and Methods

### 2.1 Study Design and Registration

This systematic review and meta‐analysis was conducted following the Preferred Reporting Items for Systematic Reviews and Meta‐Analyses (PRISMA) guidelines [21]. The study protocol was developed a priori, and the review examined the prognostic value of microsatellite instability (MSI) and Epstein–Barr virus (EBV) positivity in programmed death‐ligand 1 (PD‐L1)‐positive gastric cancer.

### 2.2 Data Sources and Search Strategy

A comprehensive literature search was performed across PubMed, EMBASE, and Cochrane Library databases, covering publications from January 2010 to December 2024. This timeframe was selected to ensure methodological consistency, as standardized PD‐L1 testing and the TCGA molecular classification became clinically relevant after 2014–2015. Additional sources included conference abstracts from ASCO, ESMO, and ASCO‐GI meetings, bibliographies of key articles, and relevant clinical trial registries (ClinicalTrials.gov).

The search strategy employed the following terms and Boolean operators:

- (“gastric cancer” OR “stomach cancer” OR “gastric adenocarcinoma” OR “gastroesophageal junction”).
- AND (“microsatellite instability” OR “MSI” OR “MSI‐H” OR “mismatch repair” OR “dMMR”).
- AND (“PD‐L1” OR “programmed death‐ligand 1” OR “CD274”).
- AND (“EBV” OR “Epstein–Barr virus” OR “EBER”).
- AND (“survival” OR “prognosis” OR “outcome” OR “mortality”).

Searches were restricted to human studies and English‐language publications.

### 2.3 Biomarker Assessment Methods

Studies were included if they assessed biomarker status using validated methodologies:

### 2.4 MSI Status

- Immunohistochemistry (IHC) for mismatch repair proteins (MLH1, MSH2, MSH6, PMS2), with loss of expression in ≥ 1 protein defining MMR‐deficient (dMMR)/MSI‐H status.
- Polymerase chain reaction (PCR)‐based microsatellite analysis using the Bethesda panel markers (BAT25, BAT26, D2S123, D5S346, D17S250) or equivalent panels.
- Next‐generation sequencing (NGS)‐based MSI assessment.

### 2.5 PD‐L1 Expression

- IHC staining using validated antibodies (22C3, 28‐8, SP142, SP263).
- Positivity defined as Combined Positive Score (CPS) ≥ 1 or Tumor Proportion Score (TPS) ≥ 1%.

### 2.6 EBV Status

- In situ hybridization (ISH) for EBV‐encoded small RNAs (EBER).
- PCR‐based EBV DNA detection.

### 2.7 Inclusion Criteria

1. Original research articles (retrospective or prospective cohort studies).
2. Patients with histologically confirmed gastric or gastroesophageal junction adenocarcinoma.
3. Assessment of MSI status, PD‐L1 expression, and/or EBV status using validated methods.
4. Reporting of survival outcomes (OS, DFS, or PFS) with hazard ratios (HRs) and 95% confidence intervals (CIs), or sufficient data for calculation.
5. English‐language publications.
6. Human studies only.

### 2.8 Exclusion Criteria

1. Preclinical studies, case reports, reviews, editorials, letters, and conference abstracts without full‐text availability.
2. Studies evaluating response to immune checkpoint inhibitors or targeted therapies without a surgery‐based or chemotherapy‐only control arm.
3. Studies in other malignancies or mixed tumor populations without separate gastric cancer data.
4. Duplicate publications or overlapping patient cohorts (most recent or largest cohort retained).
5. Studies lacking sufficient data for quantitative synthesis.

### 2.9 Definition of Disease Stage

Localized Gastric Cancer: Non‐metastatic disease (stages I–III according to AJCC/UICC TNM 7th or 8th edition staging) treated with curative‐intent surgical resection (gastrectomy with D1/D2 lymphadenectomy), with or without perioperative (neoadjuvant and/or adjuvant) chemotherapy. This category includes early gastric cancer (T1N0M0) and locally advanced resectable disease (T2‐4a, N0‐3, M0). Advanced/Metastatic Gastric Cancer: Stage IV disease or unresectable locally advanced disease treated with palliative systemic therapy.

Included studies comprised patients receiving:

- Surgery alone (curative‐intent gastrectomy).
- Surgery with perioperative chemotherapy (neoadjuvant and/or adjuvant regimens including FLOT, XELOX, SOX, S‐1‐based, or capecitabine/oxaliplatin combinations).
- Palliative chemotherapy for metastatic disease (platinum/fluoropyrimidine‐based regimens).

Studies specifically evaluating response to immune checkpoint inhibitors (pembrolizumab, nivolumab) or targeted therapies (trastuzumab, ramucirumab) were excluded to assess the prognostic (rather than predictive) value of these biomarkers.

### 2.10 Data Extraction and Quality Assessment

Data extraction was independently conducted by two reviewers (FP, MG), with discrepancies resolved by consensus or consultation with a third reviewer (AG). Extracted data included: first author, publication year, country, study design, sample size, patient demographics, disease stage, biomarker assessment methods, treatment received, follow‐up duration, and survival outcomes (HRs with 95% CIs for OS and DFS).

When HRs were not directly reported, they were calculated from Kaplan–Meier curves using established methods [22]. Study quality was assessed using the Newcastle‐Ottawa Scale (NOS) for observational studies, with scores ≥ 7 (out of 9) considered high quality.

### 2.11 Statistical Analysis

#### 2.11.1 Effect Measure

Hazard ratios (HRs) with 95% confidence intervals (CIs) were used as the primary effect measure for time‐to‐event outcomes. HR < 1 indicated improved survival in the MSI‐H/PD‐L1+ or EBV+/PD‐L1+ group compared to controls (MSS/PD‐L1− or EBV−/PD‐L1−).

#### 2.11.2 Pooling Method

Given the anticipated clinical and methodological heterogeneity inherent to gastric cancer studies (varying populations, biomarker assessment methods, treatment protocols, and geographic regions), we employed the DerSimonian‐Laird random‐effects model for all pooled analyses. This model accounts for both within‐study and between‐study variance, providing more conservative and generalizable estimates.

### 2.12 Heterogeneity Assessment

- Cochran's *Q* test (significance threshold *p* < 0.10).
- *I* ^2^ statistic: 0%–25% (low heterogeneity), 25%–50% (moderate), 50%–75% (substantial), > 75% (considerable).
- Tau^2^ for between‐study variance estimation.

#### 2.12.1 Subgroup Analyses

Pre‐specified subgroup analyses were conducted by:

- Disease stage (localized vs. metastatic).
- Outcome type (OS vs. DFS).

#### 2.12.2 Publication Bias

Assessed using funnel plot visual inspection and Egger's regression test for analyses including ≥ 10 studies.

#### 2.12.3 Software

All analyses were performed using Review Manager (RevMan) version 5.4 (Cochrane Collaboration, Copenhagen, Denmark).

## 3 Results

### 3.1 Study Selection

The systematic literature search identified 499 records from PubMed (*n* = 261) and other databases including EMBASE and Cochrane Library (*n* = 238). After removal of duplicates (*n* = 147), records marked as ineligible including reviews, case reports, and editorials (*n* = 135), and non‐English language publications (*n* = 25), 192 records underwent title and abstract screening.

During screening, 125 records were excluded because they primarily evaluated response to immunotherapy or targeted therapies. Of the 67 reports sought for full‐text retrieval, 13 were excluded for evaluating other diseases or mixed populations. Among the 54 reports assessed for eligibility, 29 were excluded due to overlapping patient cohorts or duplicate publications from the same authors (retaining the most recent or comprehensive report).

**FIGURE 1:**
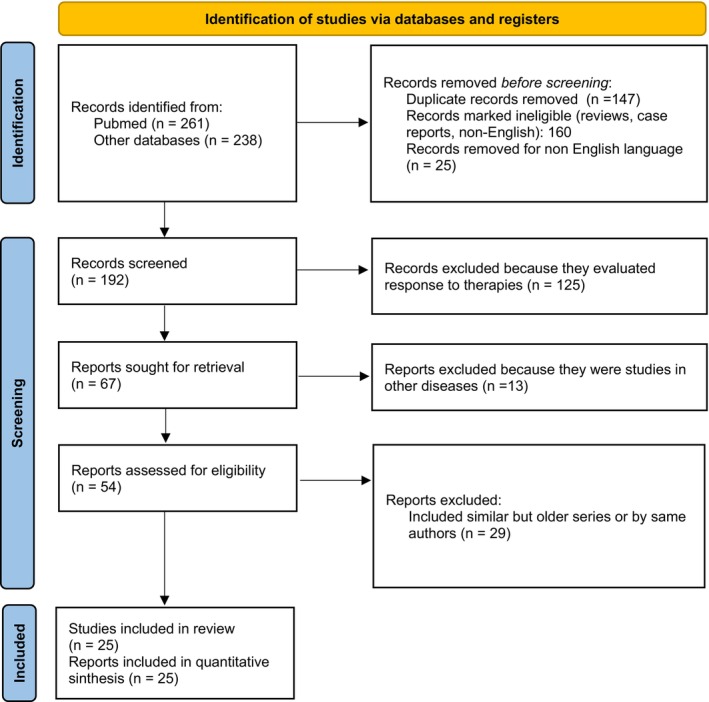
PRISMA (Preferred Reporting Items for Systematic Reviews and Meta‐Analyses) flow diagram illustrating the systematic literature search and study selection process. Initial database searches identified 499 records from PubMed (*n* = 261) and other databases including EMBASE and Cochrane Library (*n* = 238). After removal of duplicates (*n* = 147), ineligible records (*n* = 135), and non‐English publications (*n* = 25), 192 records were screened. Following exclusion of studies evaluating therapy response (*n* = 125), studies in other diseases (*n* = 13), and overlapping cohorts (*n* = 29), 25 studies met the inclusion criteria and were included in the quantitative meta‐analysis.

The final analysis included 25 studies meeting all inclusion criteria (Figure 1 and Table 1; File S1 for complete reference list of included studies).

**TABLE 1:** characteristics of included studies.

Author/year	Type of study/country	Median follow up (months)	No. of pts	Stage (localized/advanced) %	(neo)adj/palliative CT %	Surgery %	PD‐L1 (%) on TCs	MSI (%)	EBV+ (%)	Type of analysis	Bias (NOS)
Akimoto/2023	Retrosp, case–control/Japan	—	679	48.1/51.9	71.2/0	100	33.7	10.4 (dMMR)	5.1	MVA (OS)	5
Angell/2019	Retrosp/Various	—	380	35.3/64.7	—	100	16.4 (≥ 1%)	18.9 (MSI‐H)	6.6	MVA (DFS, OS)	5
Cho/2017	Retrosp/Korea	56.9	78	58.4/41.6	—	100	9	100 (MSI‐H)	0	MVA (OS)	7
Choi/2019	Retrosp/Korea	67	592	47.6/52.4	51.6/0	100	2.7	6.8 (MSI‐H)	—	MVA (DFS)	8
Choi/2020	Retrosp/Korea	77	514	70/30	—	100	19.6	10 (MSI‐H)	6	MVA (RFS, OS)	8
De Rosa/2018	Retrosp/Italy	164	169	51/49	—	100	81	86 (MSI‐H)	91	MVA (OS)	8
Di Bartolomeo/2020	Retrosp/Italy	—	256	8/92	100/0	100	11 (≥ 1%)	9 (MSI‐H)	—	MVA (DFS, OS)	5
Hagi/2020	Retrosp, cohort/Japan	—	143	0/100	—	58.7	17.4 (≥ 1%)	14.6 (dMMR)	—	MVA (PFS)	5
Hashimoto/2019	Retrosp/Japan	58.4	285	51.2/48.8	38.5/0	100	24.6 (≥ 5%)	9.8 (dMMR)	—	MVA (RFS)	7
Jin/2017	Retrosp/China	28	89	89.9/10.1	89.9/10.1	100	40.4 (≥ 1%)	32.6 (dMMR)	1.1	MVA (OS)	6
Kawazoe/2017	Retrosp/Japan	—	487	73.5/26.5	53.6/—	100	22.8 (≥ 1%)	5.1 (dMMR)	5.1	MVA (OS)	5
Kim/2020	Retrosp/Korea	—	63	0/100	—	61.9	41.3 (≥ 1%)	9.5 (MSI‐H)	6.3	MVA (PFS)	5
Kim/2022	Sub analysis of trial/Asia	28.3	45	0/100	0/100	—	36.1 (≥ 1%)	0 (dMMR)	16.7	MVA (PFS, OS)	6
Koh/2017	Retrosp/Korea	—	392	100/0	100/0	100	25.0 (≥ 10%)	9.2 (MSI‐H)		UVA (OS)	5
Kwon/2017	Retrosp/Korea	94.1	394	96.7/3.3	75/25	100	31.2 (≥ 10%)	9.4 (MSI‐H)	6.6	MVA (DFS, OS)	8
Kwon/2020	Retrosp/Korea	—	59	0/100	0/100	50.8	23.9 (≥ 1%)	16 (dMMR)	26.9	MVA (PFS)	5
Ma/2016	Retrosp/America	43.1	44	91/9	59/0	100	72 (≥ 1%)	35 (dMMR)	16	MVA (DFS)	7
Mishima/2019	Retrosp analysis/Asia	3.8	80	0/100	0/100	—	23 (≥ 1%)	10 (dMMR)	5	UVA (PFS)	5
Morihiro/2019	Retrosp/Japan	—	283	—	—	100	15.5 (≥ 5%)	7.8 (MSI‐H)	8.1	MVA (OS)	5
Namvar/2022	Retrosp/Iran	29.35	70	100/0	—	100	20 (≥ 10%)	7.1 (dMMR)	—	UVA (OS)	6
Pereira/2018	Retrosp/Brasil	36	287	100/0	68.5/31.5	100	8.8 (≥ 1%)	27 (dMMR)	10.5	UVA (DFS, OS)	7
Salati/2023	Retrosp/Western	20.9	131	20/80	0/100	28.5	31 (≥ 5%)	6.9 (dMMR)	4.3	UVA (PFS, OS)	6
Schlintl/2022	Retrosp/Austria	—	50	0/100	0/100	50	48 (≥ 1%)	16 (dMMR; MSI‐H)	—	UVA (PFS, OS)	5
Silva/2024	Retrosp/America	13.9	698	0/100	0/100	—	59.3 (≥ 1%)	4.3 (dMMR)	—	MVA (OS)	6
Yang/2021	Retrosp/China	39.7	226	100/0	100/0	—	41.2 (≥ 50%)	23.2 (dMMR; MSI‐H)	4.9	MVA (OS)	7

Abbreviations: CT, chemotherapy; DFS, disease‐free survival; dMMR, mismatch repair‐deficient; EBV, Epstein–Barr virus; MSI, microsatellite instability; MVA, multivariate analysis; OS, overall survival; PD‐L1, programmed death‐ligand 1; PFS, progression‐free survival; Retrosp, retrospective; RFS, recurrence‐free survival; TCs, tumor cells; UVA, univariate analysis.

### 3.2 Study Characteristics

The 25 included studies comprised a total of 6494 patients with gastric or gastroesophageal junction adenocarcinoma. Studies were published between 2016 and 2024, with the majority (*n* = 18) originating from East Asian countries (China, Japan, South Korea) and the remainder from Western countries (Italy, Germany, United States, Brazil). Sample sizes ranged from 45 to 1058 patients per study.

MSI status was assessed by IHC for MMR proteins in 15 studies, PCR‐based analysis in 7 studies, and NGS in 3 studies. PD‐L1 expression was evaluated using the 22C3 antibody in 12 studies, SP263 in 6 studies, and other validated antibodies in 7 studies. EBV status was determined by EBER‐ISH in all studies reporting EBV data.

### 3.3 MSI‐H/PD‐L1+ and Survival Outcomes

#### 3.3.1 Overall Survival (OS)

**FIGURE 2:**
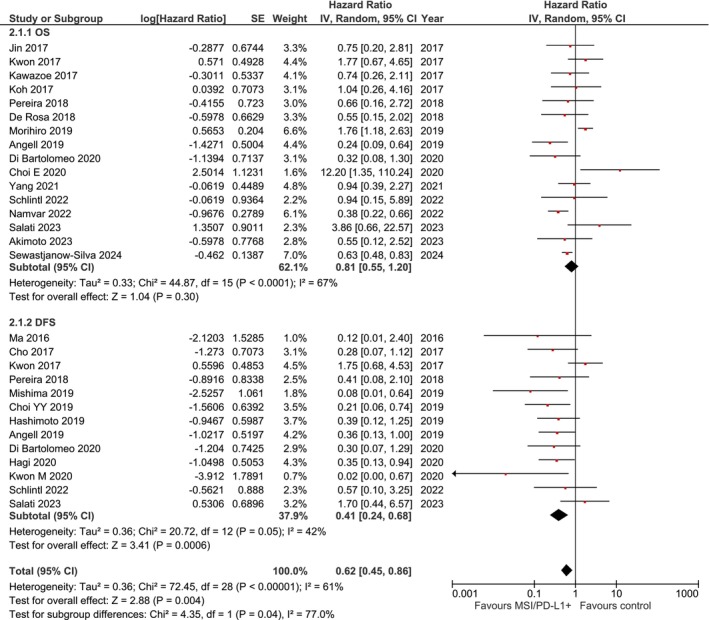
Forest plot depicting the association between MSI‐high/PD‐L1‐positive status and survival outcomes in gastric cancer patients. Panel A (2.1.1): Overall survival (OS) analysis including 13 studies. The pooled hazard ratio (HR) was 0.78 (95% CI: 0.48–1.28; *p* = 0.33), indicating no statistically significant difference. Heterogeneity was substantial (*I* ^2^ = 69%; *p* < 0.001; Tau^2^ = 0.49). Panel B (2.1.2): Disease‐free survival (DFS) analysis including 8 studies. The pooled HR was 0.42 (95% CI: 0.23–0.75; *p* = 0.004), demonstrating significantly improved DFS in MSI‐H/PD‐L1+ patients. Heterogeneity was moderate (*I* ^2^ = 36%; *p* = 0.14; Tau^2^ = 0.25). Analysis performed using random‐effects model (DerSimonian‐Laird method). Effect sizes are displayed as squares (proportional to study weight) with 95% confidence intervals (horizontal lines). Diamond represents pooled estimate. CI, confidence interval; HR, hazard ratio; IV, inverse variance; SE, standard error.

Thirteen studies reported OS data for MSI‐H/PD‐L1+ versus MSS/PD‐L1− gastric cancer patients. The pooled analysis demonstrated no statistically significant difference in OS (HR, 0.78; 95% CI, 0.48–1.28; *p* = 0.33) (Figure 2, Panel A). Heterogeneity was substantial (*I* ^2^ = 69%; *p* < 0.001; Tau^2^ = 0.49), reflecting variability in study populations, treatment protocols, and follow‐up durations.

#### 3.3.2 Disease‐Free Survival (DFS)

Eight studies reported DFS data for patients with localized gastric cancer. MSI‐H/PD‐L1+ status was associated with significantly improved DFS compared to MSS/PD‐L1− tumors (HR, 0.42; 95% CI, 0.23–0.75; *p* = 0.004) (Figure 2, Panel B). Heterogeneity was moderate (*I* ^2^ = 36%; *p* = 0.14; Tau^2^ = 0.25).

The combined analysis of OS and DFS demonstrated an overall HR of 0.63 (95% CI, 0.42–0.93; *p* = 0.02) favoring MSI‐H/PD‐L1+ tumors, with substantial heterogeneity (*I* ^2^ = 64%; *p* < 0.0001).

### 3.4 EBV+/PD‐L1+ and Survival Outcomes

#### 3.4.1 Overall Survival (OS)

**FIGURE 3:**
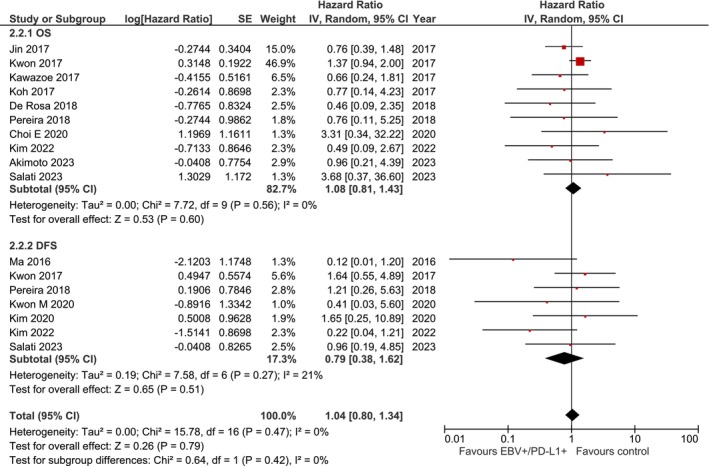
Forest plot depicting the association between EBV‐positive/PD‐L1‐positive status and survival outcomes in gastric cancer patients. Panel A (2.2.1): Overall survival (OS) analysis including 8 studies. The pooled hazard ratio (HR) was 1.08 (95% CI: 0.81–1.45; *p* = 0.59), indicating no statistically significant difference. Heterogeneity was absent (*I* ^2^ = 0%; *p* = 0.56; Tau^2^ = 0.00). Panel B (2.2.2): Disease‐free survival (DFS) analysis including 4 studies. The pooled HR was 0.81 (95% CI: 0.28–2.34; *p* = 0.70), showing no significant difference. Heterogeneity was moderate (*I* ^2^ = 34%; *p* = 0.21; Tau^2^ = 0.40). Analysis performed using random‐effects model (DerSimonian‐Laird method). Effect sizes are displayed as squares (proportional to study weight) with 95% confidence intervals (horizontal lines). Diamond represents pooled estimate. CI, confidence interval; EBV, Epstein–Barr virus; HR, hazard ratio; IV, inverse variance; SE, standard error.

Eight studies reported OS data for EBV+/PD‐L1+ versus EBV−/PD‐L1− gastric cancer patients. The pooled analysis showed no significant difference in OS (HR, 1.08; 95% CI, 0.81–1.45; *p* = 0.59) (Figure 3, Panel A). Notably, heterogeneity was absent (*I* ^2^ = 0%; *p* = 0.56; Tau^2^ = 0.00), indicating consistent findings across studies.

#### 3.4.2 Disease‐Free Survival (DFS)

Four studies reported DFS data for EBV+/PD‐L1+ patients. No significant difference was observed compared to EBV−/PD‐L1− cases (HR, 0.81; 95% CI, 0.28–2.34; *p* = 0.70) (Figure 3, Panel B). Heterogeneity was moderate (*I* ^2^ = 34%; *p* = 0.21; Tau^2^ = 0.40).

The combined analysis yielded an overall HR of 1.07 (95% CI, 0.82–1.41; *p* = 0.62) with no heterogeneity (*I* ^2^ = 0%; *p* = 0.50).

### 3.5 Advanced/Metastatic Disease

For patients with advanced or metastatic gastric cancer, neither MSI‐H nor EBV‐positive status demonstrated significant prognostic effects on survival outcomes when treated with conventional chemotherapy regimens.

## 4 Discussion

This systematic review and meta‐analysis evaluated the prognostic significance of MSI, EBV positivity, and PD‐L1 co‐expression in gastric cancer patients treated with surgery and/or conventional chemotherapy. Our findings demonstrate that while MSI‐H status is associated with improved DFS in localized disease (HR, 0.42; *p* = 0.004), neither MSI‐H nor EBV positivity significantly impacts OS in gastric cancer patients not receiving immunotherapy.

The TCGA comprehensive molecular characterization of gastric adenocarcinoma established four distinct molecular subtypes: EBV‐positive (9%), MSI‐H (22%), genomically stable (20%), and chromosomally unstable (50%) [7]. While this classification has revolutionized our understanding of gastric cancer biology and identified potential therapeutic targets, the original TCGA analysis did not demonstrate significant survival differences among subtypes [7]. Our meta‐analysis corroborates these findings, suggesting that molecular subtyping based on MSI and EBV status has limited prognostic utility independent of treatment selection.

The prevalence of PD‐L1 positivity varies by disease stage, with reported rates of 27% in resectable localized disease (stages I–II) increasing to 41% in stage III–IV disease [11]. MSI‐H and EBV‐positive tumors demonstrate higher PD‐L1 positivity rates of approximately 50%–60%, reflecting their immunogenic tumor microenvironment [14, 15, 16]. Despite this association, our analysis indicates that PD‐L1 co‐expression does not confer additional prognostic information beyond MSI or EBV status alone.

The improved DFS observed in MSI‐H patients with localized disease aligns with previous reports suggesting that MSI‐H tumors may have a more favorable natural history, potentially due to enhanced immune surveillance [23]. However, the lack of OS benefit suggests that this advantage may be attenuated over time, possibly due to disease recurrence patterns or subsequent treatment effects.

Several factors may explain the absence of clear prognostic differences among molecular subtypes. First, gastric cancer exhibits substantial intratumoral heterogeneity, particularly in MMR protein expression and EBV distribution, which may lead to misclassification and dilute prognostic associations [24]. Second, the included studies employed varied methodologies for biomarker assessment—different antibodies for PD‐L1 IHC (22C3, 28‐8, SP142, SP263), diverse scoring criteria (CPS vs. TPS), and alternative testing methods for MSI (IHC, PCR, NGS) and EBV (ISH, PCR). These methodological inconsistencies likely contributed to variability in reported results.

Geographic and ethnic factors also influence the prevalence and prognostic impact of these biomarkers. Previous meta‐analyses predominantly included East Asian studies, where gastric cancer incidence, molecular characteristics, and treatment approaches differ from Western populations [25]. By including both Eastern and Western studies, our analysis provides a broader perspective but also highlights regional differences that may influence outcomes.

It is crucial to distinguish between prognostic and predictive biomarker roles. While our analysis demonstrates limited prognostic value for MSI and EBV status, these biomarkers have established predictive utility for immunotherapy response. MSI‐H gastric cancers demonstrate remarkable responses to immune checkpoint inhibitors, with objective response rates of 40%–60% and durable remissions in a subset of patients [18]. Similarly, EBV‐positive tumors, with their high PD‐L1 expression and immune infiltration, represent promising candidates for immunotherapy [26].

This meta‐analysis has several limitations. First, the included studies were predominantly retrospective, introducing potential selection and information biases. Second, heterogeneity in biomarker assessment methods limits the ability to establish standardized thresholds. Third, the exclusion of immunotherapy studies, while necessary to assess prognostic value, limits generalizability to contemporary treatment paradigms. Finally, publication bias cannot be excluded, although funnel plot analysis did not suggest significant asymmetry.

This systematic review and meta‐analysis underscores the nuanced role of MSI and EBV as biomarkers in gastric cancer. While MSI‐H status was associated with improved DFS in localized disease, its lack of prognostic significance for OS suggests a limited role beyond guiding immunotherapy decisions. Similarly, EBV positivity, despite its strong association with PD‐L1 expression, did not translate into significant survival benefits in patients treated with surgery and conventional chemotherapy. These findings highlight the need for robust biomarker standardization and emphasize that the primary clinical utility of MSI and EBV testing lies in identifying candidates for immune checkpoint inhibitor therapy rather than prognostic stratification.

## Author Contributions

**Fausto Petrelli:** conceptualization, investigation, writing – original draft, methodology, writing – review and editing, software, formal analysis, data curation, validation. **Maria Antista:** investigation, writing – review and editing. **Antonio Ghidini:** methodology, validation, writing – review and editing. **Valentina Rampulla:** investigation, writing – review and editing. **Lorenzo Dottorini:** investigation, writing – review and editing. **Andrea Celotti:** validation. **Fulvia Milena Cribiu:** investigation, writing – review and editing. **Barbara Galassi:** investigation, writing – review and editing. **Ornella Garrone:** investigation, writing – review and editing. **Alberto Zaniboni:** writing – review and editing. **Gianluca Tomasello:** investigation, writing – review and editing. **Michele Ghidini:** conceptualization, methodology, investigation, data curation, writing – original draft, writing – review and editing.

## Funding

The authors have nothing to report.

## Conflicts of Interest

The authors declare no conflicts of interest.

## Supporting information


**Data S1:** References of included studies.

## Data Availability

No new data were generated in this study. All data analyzed are derived from published articles and/or publicly available sources cited in the manuscript and Supporting Information.

## References

[1] H. Sung , J. Ferlay , R. L. Siegel , et al., “Global Cancer Statistics 2020: GLOBOCAN Estimates of Incidence and Mortality Worldwide for 36 Cancers in 185 Countries,” CA: A Cancer Journal for Clinicians 71, no. 3 (2021): 209–249, 10.3322/caac.21660. 33538338

[2] E. C. Smyth , M. Nilsson , H. I. Grabsch , N. C. van Grieken , and F. Lordick , “Gastric Cancer,” Lancet 396, no. 10251 (2020): 635–648, 10.1016/S0140-6736(20)31288-5. 32861308

[3] F. Lordick , F. Carneiro , S. Cascinu , et al., “Gastric Cancer: ESMO Clinical Practice Guideline for Diagnosis, Treatment and Follow‐Up,” Annals of Oncology 33, no. 10 (2022): 1005–1020, 10.1016/j.annonc.2022.07.004. 35914639

[4] K. Polom , L. Marano , D. Marrelli , et al., “Meta‐Analysis of Microsatellite Instability in Relation to Clinicopathological Characteristics and Overall Survival in Gastric Cancer,” British Journal of Surgery 105, no. 3 (2018): 159–167, 10.1002/bjs.10663. 29091259

[5] Y. Y. Choi , J. M. Bae , J. Y. An , et al., “Is Microsatellite Instability a Prognostic Marker in Gastric Cancer? A Systematic Review With Meta‐Analysis,” Journal of Surgical Oncology 110, no. 2 (2014): 129–135, 10.1002/jso.23618. 24737677

[6] F. Pietrantonio , R. Miceli , A. Raimondi , et al., “Individual Patient Data Meta‐Analysis of the Value of Microsatellite Instability as a Biomarker in Gastric Cancer,” Journal of Clinical Oncology 37, no. 35 (2019): 3392–3400, 10.1200/JCO.19.01124. 31513484

[7] Cancer Genome Atlas Research Network , “Comprehensive Molecular Characterization of Gastric Adenocarcinoma,” Nature 513, no. 7517 (2014): 202–209, 10.1038/nature13480. 25079317 PMC4170219

[8] R. Giampieri , E. Maccaroni , A. Mandolesi , et al., “Mismatch Repair Deficiency May Affect Clinical Outcome Through Immune Response Activation in Metastatic Gastric Cancer Patients Receiving First‐Line Chemotherapy,” Gastric Cancer 20, no. 1 (2017): 156–163, 10.1007/s10120-016-0594-4. 26796888

[9] G. Murphy , R. Pfeiffer , M. C. Camargo , and C. S. Rabkin , “Meta‐Analysis Shows That Prevalence of Epstein‐Barr Virus‐Positive Gastric Cancer Differs Based on Sex and Anatomic Location,” Gastroenterology 137, no. 3 (2009): 824–833, 10.1053/j.gastro.2009.05.001. 19445939 PMC3513767

[10] H. Dong , S. E. Strome , D. R. Salomao , et al., “Tumor‐Associated B7‐H1 Promotes T‐Cell Apoptosis: A Potential Mechanism of Immune Evasion,” Nature Medicine 8, no. 8 (2002): 793–800, 10.1038/nm730. 12091876

[11] M. Zhang , Y. Dong , H. Liu , et al., “The Clinicopathological and Prognostic Significance of PD‐L1 Expression in Gastric Cancer: A Meta‐Analysis of 10 Studies With 1,901 Patients,” Scientific Reports 6 (2016): 37933, 10.1038/srep37933. 27892511 PMC5124943

[12] L. Gu , M. Chen , D. Guo , et al., “PD‐L1 and Gastric Cancer Prognosis: A Systematic Review and Meta‐Analysis,” PLoS One 12, no. 8 (2017): e0182692, 10.1371/journal.pone.0182692. 28796808 PMC5552131

[13] J. W. Kim , K. H. Nam , S. H. Ahn , et al., “Prognostic Implications of Immunosuppressive Protein Expression in Tumors as Well as Immune Cell Infiltration Within the Tumor Microenvironment in Gastric Cancer,” Gastric Cancer 19, no. 1 (2016): 42–52, 10.1007/s10120-014-0440-5. 25424150

[14] S. Derks , X. Liao , A. M. Chiaravalli , et al., “Abundant PD‐L1 Expression in Epstein‐Barr Virus‐Infected Gastric Cancers,” Oncotarget 7, no. 22 (2016): 32925–32932, 10.18632/oncotarget.9076. 27147580 PMC5078063

[15] S. De Rosa , N. Sahnane , M. G. Tibiletti , et al., “EBV+ and MSI Gastric Cancers Harbor High PD‐L1/PD‐1 Expression and High CD8+ Intratumoral Lymphocytes,” Cancers (Basel) 10, no. 4 (2018): 102, 10.3390/cancers10040102. 29614789 PMC5923357

[16] C. Böger , H. M. Behrens , M. Mathiak , S. Krüger , H. Kalthoff , and C. Röcken , “PD‐L1 Is an Independent Prognostic Predictor in Gastric Cancer of Western Patients,” Oncotarget 7, no. 17 (2016): 24269–24283, 10.18632/oncotarget.8169. 27009855 PMC5029700

[17] C. S. Fuchs , T. Doi , R. W. Jang , et al., “Safety and Efficacy of Pembrolizumab Monotherapy in Patients With Previously Treated Advanced Gastric and Gastroesophageal Junction Cancer: Phase 2 Clinical KEYNOTE‐059 Trial,” JAMA Oncology 4, no. 5 (2018): e180013, 10.1001/jamaoncol.2018.0013. 29543932 PMC5885175

[18] Y. Y. Janjigian , K. Shitara , M. Moehler , et al., “First‐Line Nivolumab Plus Chemotherapy Versus Chemotherapy Alone for Advanced Gastric, Gastro‐Oesophageal Junction, and Oesophageal Adenocarcinoma (CheckMate 649): A Randomised, Open‐Label, Phase 3 Trial,” Lancet 398, no. 10294 (2021): 27–40, 10.1016/S0140-6736(21)00797-2. 34102137 PMC8436782

[19] N. Yang , Y. Wu , M. Jin , et al., “Microsatellite Instability and Epstein‐Barr Virus Combined With PD‐L1 Could Serve as a Potential Strategy for Predicting the Prognosis and Efficacy of Postoperative Chemotherapy in Gastric Cancer,” PeerJ 9 (2021): e11481, 10.7717/peerj.11481. 34046266 PMC8139270

[20] M. C. Camargo , W. H. Kim , A. M. Chiaravalli , et al., “Improved Survival of Gastric Cancer With Tumour Epstein‐Barr Virus Positivity: An International Pooled Analysis,” Gut 63, no. 2 (2014): 236–243, 10.1136/gutjnl-2013-304531. 23580779 PMC4384434

[21] M. J. Page , J. E. McKenzie , P. M. Bossuyt , et al., “The PRISMA 2020 Statement: An Updated Guideline for Reporting Systematic Reviews,” BMJ (Clinical Research Ed.) 372 (2021): n71, 10.1136/bmj.n71. PMC8005924 33782057

[22] J. F. Tierney , L. A. Stewart , D. Ghersi , S. Burdett , and M. R. Sydes , “Practical Methods for Incorporating Summary Time‐To‐Event Data Into Meta‐Analysis,” Trials 8 (2007): 16, 10.1186/1745-6215-8-16. 17555582 PMC1920534

[23] E. C. Smyth , A. Wotherspoon , C. Peckitt , et al., “Mismatch Repair Deficiency, Microsatellite Instability, and Survival: An Exploratory Analysis of the Medical Research Council Adjuvant Gastric Infusional Chemotherapy (MAGIC) Trial,” JAMA Oncology 3, no. 9 (2017): 1197–1203, 10.1001/jamaoncol.2016.6762. 28241187 PMC5824280

[24] M. Ratti , A. Lampis , J. C. Hahne , R. Passalacqua , and N. Valeri , “Microsatellite Instability in Gastric Cancer: Molecular Bases, Clinical Perspectives, and New Treatment Approaches,” Cellular and Molecular Life Sciences 75, no. 22 (2018): 4151–4162, 10.1007/s00018-018-2906-9. 30173350 PMC6182336

[25] S. J. Lin , J. A. Gagnon‐Bartsch , I. B. Tan , et al., “Signatures of Tumour Immunity Distinguish Asian and Non‐Asian Gastric Adenocarcinomas,” Gut 64, no. 11 (2015): 1721–1731, 10.1136/gutjnl-2014-308252. 25385008 PMC4680172

[26] S. T. Kim , R. Cristescu , A. J. Bass , et al., “Comprehensive Molecular Characterization of Clinical Responses to PD‐1 Inhibition in Metastatic Gastric Cancer,” Nature Medicine 24, no. 9 (2018): 1449–1458, 10.1038/s41591-018-0101-z. 30013197

