# Supplementary material for: Prognostic Significance of MSI and EBV Positivity in PD‐L1 Positive Gastric Cancer: A Systematic Review and Meta‐Analysis
Source: Cancer Med. 2026 Mar 19;15(3):e71711. doi: 10.1002/cam4.71711 (PMC13093534; doi:10.1002/cam4.71711)
Supplement: Supplementary file 1 — Data S1: References of included studies. [file CAM4-15-e71711-s001.docx]

**References of included studies**

1. Akimoto T, Furuya T, Murakami K, et al. Immune landscape of gastric cancer: PD-L1 expression and its correlation with tumor-infiltrating lymphocytes and clinicopathological factors. Int J Clin Oncol. 2023;28(4):1234-1245. doi:10.1007/s10147-023-02174-8​
2. Angell H, Galon J. Programmatic death-ligand 1 expression, immune contexture, and the prognostic and predictive impact in cancer. Oncoimmunology. 2019;8(3):e1515615. doi:10.1080/2162402X.2018.1515615​
3. Cho J, Lee J, Bang H, et al. Programmed death-ligand 1 expression predicts survival in patients with gastric carcinoma with microsatellite instability. Oncotarget. 2017;8(8):13320-13328. doi:10.18632/oncotarget.14698​
4. Choi E, Chang MS, Byeon SJ, et al. Prognostic perspectives of PD-L1 combined with tumor-infiltrating lymphocytes, Epstein-Barr virus, and microsatellite instability in gastric carcinomas. Diagn Pathol. 2020;15:69. doi:10.1186/s13000-020-00979-z​
5. Choi YY, Kim H, Shin SJ, et al. Microsatellite instability and programmed cell death ligand 1 expression in stage II/III gastric cancer: A post hoc analysis of the CLASSIC trial. Ann Surg. 2019;270(2):309-316. doi:10.1097/SLA.0000000000002803​
6. De Rosa V, Toitiello F, et al. EBV-associated gastric cancer: Molecular subtypes and therapeutic approaches. Oncotarget. 2018;9(24):17252-17267. doi:10.18632/oncotarget.24678 .
7. Di Bartolomeo M, Pietraellegrinelli A, et al. PD-L1 expression and its role in the treatment of EBV and MSI gastric cancers. Ther Adv Med Oncol. 2020;12:1758835920913482. doi:10.1177/1758835920913482 .
8. Hagi T, Takashima A, Kuwata T, etle of mismatch repair deficiency and immune checkpoint inhibitors in gastric cancer treatment. J Gastroenterol Hepatol. 2020;35(6):996-1006. doi:10.1111/jgh.14936 .
9. Hashimoto T, Kurokawa Y, Takahashi T, et al. Predictive value of MLH1 and PD-L1 expression for prognosis and response to preoperative chemotherapy in gastric cancer. Gastric Cancer. 2019;22:785-792. doi:10.1007/s10120-018-00918-4​
10. Jin S, Xu B, Yu L, et al. PD-1, PD-L1 expression, and CD3+ T cell infiltration in advanced gastric signet-ring cell carcinoma: Potential biomarkers for immunotherapy. Oncotarget. 2017;8(24):38850-38862. doi:10.18632/oncotarget.16939​
11. Kawazoe A, Kuwata T, Kuboki Y, et al. Clinicopathological features of programmed death ligand 1 expression with tumor-infiltrating lymphocytes, mismatch repair, and Epstein–Barr virus status in gastric cancer. Gastric Cancer. 2017;20:407-415. doi:10.1007/s10120-016-0631-3​
12. Kim J, Kim B, Kang SY, et al. Tumor mutational burden determined by panel sequencing predicts survival after immunotherapy in patients with advanced gastric cancer. Front Oncol. 2020;10:314. doi:10.3389/fonc.2020.00314​
13. Kim JH, Ryu MH, Park YS, et al. Predictive biomarkers for the efficacy of nivolumab as ≥3rd-line therapy in patients with advanced gastric cancer: A subset analysis of ATT RAC TION-2 phase III trial. BMC Cancer. 2022;22:378. doi:10.1186/s12885-022-09488-2​
14. Koh J, Ock CY, Kim JW, et al. Clinicopathologic implications of immune classification by PD-L1 expression and CD8-positive tumor-infiltrating lymphocytes in gastric cancer patients. Oncotarget. 2017;8(16):26356-26367. doi:10.18632/oncotarget.15541​
15. Kwon M, Hong JY, Kim ST, et al. Association of serine/threonine kinase 11 mutations and response to programmed cell death 1 inhibitors in metastatic gastric cancer. Pathol Res Pract. 2020;216:152947. doi:10.1016/j.prp.2020.152947​
16. Kwon MJ, Kim KC, Nam ES, et al. Programmed death ligand-1 and MET co-expression is a poor prognostic factor in gastric cancers after resection. Oncotarget. 2017;8(47):82399-82414. doi:10.18632/oncotarget.20750​
17. Ma C, Patel K, Singhi AD, et al. Programmed Death-Ligand 1 Expression Is Common in Gastric Cancer Associated With Epstein-Barr Virus or Microsatellite Instability. Am J Surg Pathol. 2016;40(11):1496-1506. doi:10.1097/PAS.0000000000000711​
18. Mishima S, Kawazoe A, Nakamura Y, et al. Clinicopathological and molecular features of responders to nivolumab for patients with advanced gastric cancer. J Immunother Cancer. 2019;7:24. doi:10.1186/s40425-019-0514-3​
19. Morihiro T, Kuroda S, Kanaya N, et al. PD-L1 expression combined with microsatellite instability/CD8+ tumor infiltrating lymphocytes as a useful prognostic biomarker in gastric cancer. Sci Rep. 2019;9:4633. doi:10.1038/s41598-019-41177-2​
20. Namvar N, Montazer M, Ahmadvand S, et al. Expression of PD-1 in Tumor Cells is Associated with Shorter Survival in Non-metastatic Intestinal-type Gastric Adenocarcinoma. Iran J Allergy Asthma Immunol. 2022;21(6):600-615. doi:10.18502/ijaai.v21i6.11519​
21. Pereira MA, Ramos MFKP, Faraj SF, et al. Clinicopathological and prognostic features of Epstein-Barr virus infection, microsatellite instability, and PD-L1 expression in gastric cancer. J Surg Oncol. 2018;117(5):829-839. doi:10.1002/jso.25022​
22. Salati M, Ghidini M, Paccagnella M, et al. Clinical significance of molecular subtypes in Western advanced gastric cancer: A real-world multicenter experience. Int J Mol Sci. 2023;24(1):813. doi:10.3390/ijms24010813​
23. Schlintl V, Huemer F, Rinnerthaler G, et al. Checkpoint inhibitors in metastatic gastric and GEJ cancer: A multi-institutional retrospective analysis of real-world data in a Western cohort. BMC Cancer. 2022;22:51. doi:10.1186/s12885-021-09115-6​
24. Silva MS, Kwiatkowski E, Yamashita K, et al. Three biomarkers (HER2, PD-L1, and microsatellite status) in a large cohort of metastatic gastroesophageal adenocarcinomas: The MD Anderson Cancer Center experience. Int J Cancer. 2024;155(12):2277-2286. doi:10.1002/ijc.35090​
25. Yang N, Wu Y, Jin M, et al. Microsatellite instability and Epstein-Barr virus combined with PD-L1 could serve as a potential strategy for predicting the prognosis and efficacy of postoperative chemotherapy in gastric cancer. PeerJ. 2021;9:e11481. doi:10.7717/peerj.11481​
